# The Relationship Between Air Pollution, Meteorological Factors, and Eosinophil Counts in Peripheral Blood of Patients With Allergic Rhinitis: A Cross-Sectional Study

**DOI:** 10.1155/carj/4709567

**Published:** 2025-02-20

**Authors:** Boya Fan, Gang Wang, Lei Wang, Wei Wu

**Affiliations:** ^1^Department of Otolaryngology Head and Neck Surgery, The Ninth Medical Center of PLA General Hospital, Beijing 100101, China; ^2^Department of Otolaryngology Head and Neck Surgery, Peking University Third Hospital, Beijing 100191, China; ^3^State Environmental Protection Key Laboratory of Environmental Sense Organ Stress and Health, Beijing 100101, China

**Keywords:** air pollution, allergic rhinitis, eosinophil, meteorological factors

## Abstract

**Objective:** To evaluate the relationship between air pollution, meteorological factors, and eosinophils in the peripheral blood of allergic rhinitis (AR) patients.

**Methods:** We conducted a retrospective study of medical records from the Ninth Medical Center of PLA General Hospital in Beijing. A review of medical records (1^st^ January 2014–31^th^ August 2022) of 1080 AR patients who underwent peripheral blood eosinophil count tests. Patients were stratified into elevated and normal eosinophil count groups. Daily meteorological data (mean temperature and relative humidity) and ambient pollutant levels (PM2.5, PM10, NO_2_, SO_2_, CO, and ozone [O_3_]) were collected. The mean pollutant levels and meteorological factors on the day of eosinophil count measurement were calculated separately for each group. Linear regression was performed to analyze the association between eosinophil counts and both meteorological factors and pollutant levels among AR patients.

**Results:** In 1080 AR patients, 11.85% had elevated eosinophil counts. Higher temperature (16.88 ± 9.09°C), humidity (57.75 ± 17.22%), and O_3_ levels (116.54 ± 54.92 μg/m^3^) correlated significantly (*p* < 0.05) with elevated eosinophil counts. Linear regression confirmed positive associations between eosinophil count and temperature (*β* = 0.003), humidity (*β* = 0.001), and O_3_ (*β* = 0.0004) (*p* < 0.05). Other pollutants showed no significant differences.

**Conclusion:** Elevated eosinophil counts in AR patients correlated significantly with higher temperature, humidity, and O_3_ levels. Linear regression confirmed positive associations between eosinophil count and these meteorological factors.

## 1. Introduction

Allergic rhinitis (AR) is one of the most common upper respiratory tract chronic inflammation, developing due to patient allergen exposure. Approximately 500 million people worldwide suffer from this disease, with particularly high numbers in Europe and the United States [1]. The prevalence of AR ranges from 5% to 50% and has been increasing globally [2]. In recent years, as China's economy has rapidly developed and industrial facilities have improved, the number of patients with AR in that country has been increasing, and various discussions about the disease are becoming increasingly intense [3]. The high incidence rate of AR has brought a burden on human health and has a significant financial impact on the direct and indirect costs of the disease [4–6].

AR is a type I hypersensitivity reaction triggered by heightened sensitivity of the nasal mucosa to environmental allergens, primarily mediated through immunoglobulin E (IgE) receptors. This process is driven by an imbalance in *T*-helper cell responses, specifically an overactivation of Th2 cells, which play a critical role in AR. In individuals with AR, Th2 cells release cytokines such as IL-4, IL-5, and IL-13, which promote IgE production and eosinophil recruitment, leading to inflammation and allergic symptoms in the nasal cavity [7]. Usually, due to the stimulation of allergens, allergic reactions occur in the nasal mucosa, including hyperemia, edema, and exudation of inflammatory secretions. They can lead to some common symptoms in AR patients, including sneezing, nasal congestion, nasal itching, and runny nose [8]. Typically, high levels of eosinophils are detected in the peripheral blood of AR patients and eosinophils could play an active role in the immune response after exposure to allergens [9]. There is evidence that eosinophilia in peripheral blood is a characteristic of AR [9–11]. However, at the same time, the impact of environmental pollution caused by the highly developed industrial economy on it and the changes caused by different weather conditions cannot be ignored. Among various pollutants, particulate is one of the most representative and harmful things.

Research on the impact of climate change on respiratory allergies is still lacking, and epidemiological studies on the relationship between inflammatory responses and environmental factors (such as meteorological variables and air pollution) in patients with AR still require a large amount of patient information to provide more explanations. Prior research has demonstrated that air pollution and various meteorological parameters have an impact on the levels of allergens present, which in turn can influence the development of AR. These factors can alter the biological makeup and content of airborne allergens, such as pollen, and alter their dispersion and concentration. In particular, air pollution may cause the release of allergenic particles or enhance their binding to pollutant particles. In addition, meteorological factors such as temperature, humidity, and wind speed can affect pollen release and dispersion [12]. The elevation of eosinophils, a type of white blood cell, is often observed in patients with allergic rhinitis and can be an important clue in its diagnosis. When exposed to allergens, the immune system of susceptible individuals may exhibit increased production of immunoglobulin E (IgE) antibodies, hypersensitivity reactions that release histamine and other inflammatory mediators, and accumulation of eosinophils in the nasal mucosa. The measurement of blood eosinophil counts or nasal eosinophil counts may be useful in the diagnosis of AR and monitoring the response to treatment [13].

Consequently, recognition and understanding the relationship between these environmental factors and the onset of AR or the role of eosinophils in AR can be crucial in effective management and prevention of this disease. Further research is warranted to elucidate the precise mechanisms involved and to develop strategies to mitigate their impact on public health. This study focused on investigating the impact of atmospheric pollution and meteorological factors on peripheral blood eosinophil levels in AR patients.

## 2. Method

### 2.1. Study Population

This retrospective cross-sectional study encompassed patients diagnosed with AR in the Otolaryngology Department of the Ninth Medical Center of PLA General Hospital in Beijing, between January 1st, 2014, and August 31st, 2022. All patients included in this study were residents of Beijing. Patient data included demographic details such as gender, age, address, date of hospital visit, and disease diagnosis. Population statistics, clinical parameters, dates, and geographical locations related to AR were collected. Laboratory results obtained on admission day, including peripheral blood eosinophil counts, were recorded. A total of 1080 cases of AR across all age groups were identified, based on the International Classification of Diseases (ICDs, 10th Revision) codes for AR (J30.401) and clinical diagnoses by physicians, specifically including individuals who had undergone peripheral blood eosinophil count assessments. The exclusion criteria were as follows: (1) any infectious diseases within the 2 weeks preceding the blood cell analysis; (2) abnormal liver or kidney function; and (3) presence of viral infections or positive carrier status (Hepatitis B virus, syphilis, and HIV/AIDS). Subsequently, patients were categorized based on elevated peripheral blood eosinophil counts (> 0.52 × 10^9^/L) or normal counts. This study was conducted following the principles of the Helsinki Declaration. Ethical approval was granted by the Ethics Committee of the Ninth Medical Center of PLA General Hospital.

### 2.2. Air Pollution and Meteorological Data

Data on ambient air pollution spanning from 2014 to 2022 were acquired from the Beijing Municipal Environmental Monitoring Center, utilizing information from 35 monitoring stations uniformly dispersed across the entirety of Beijing. Specifically, we selected a monitoring station situated at the Olympic Sports Center in Chaoyang District, Beijing, strategically located within a proximity of fewer than 3 km from the hospital. Hourly concentrations of pollutants including PM2.5 (μg/m^3^), PM10 (μg/m^3^), NO_2_ (μg/m^3^), SO_2_ (μg/m^3^), CO (mg/m^3^), and O_3_ (μg/m^3^) were obtained from this station. Daily concentrations for these pollutants were computed based on measurements that were valid for a minimum of 18–24 h per day. These daily air pollutant concentrations served as the baseline for assessing the overall health impact. In addition, data regarding daily ambient temperature (°C) and relative humidity (%) in Beijing during the same time frame were sourced from the China Meteorological Data Sharing Service System (https://data.cma.cn/).

### 2.3. Statistical Analysis

For statistical analyses, the *R* (4.2.1) and GraphPad Prism 9 were used. Categorical variables will be presented using frequencies and percentages, while continuous variables will be expressed as the mean ± standard deviation. The differences in continuous variables were analyzed using the independent sample *t*-test for normally distributed variables. The threshold for statistical significance was established at a two-tailed *p* value below 0.05. Linear regression was employed to model the association between the peripheral blood eosinophil count and the corresponding meteorological factors and pollutant levels measured on the same day among patients with AR.

## 3. Result

Among 1080 eligible patients (475 females (43.98%); mean (SD) age of 32.80 (19.70) years), 128 individuals (11.85%) exhibited an elevated peripheral blood eosinophil count, exceeding the normal value (> 0.52 × 10^9^/L), whereas 952 cases (88.15%) demonstrated a normal peripheral blood eosinophil count (Table 1). Of the 128 patients with elevated eosinophil counts, 75 were male (58.59%) and 53 were female (41.41%). Among the 952 patients with normal eosinophil counts, 530 were male (55.67%) and 422 were female (44.33%). The mean (SD) peripheral blood eosinophil count was 0.25 (0.23) in AR patients.

**Figure 1:**
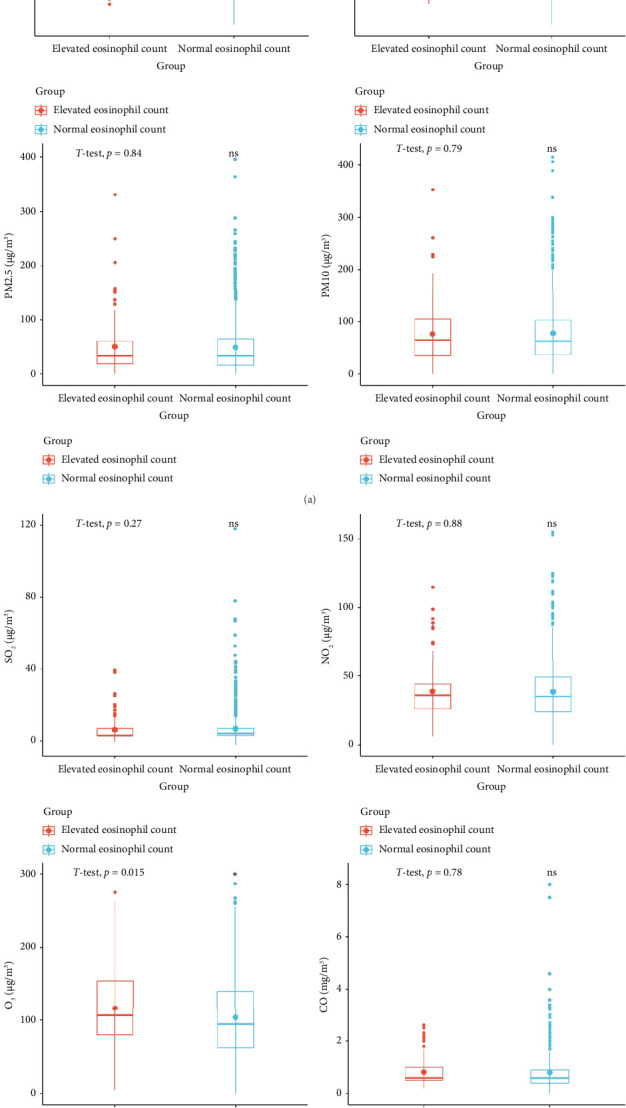
The box diagrams show the situation of meteorological factors and pollutants when eosinophils are elevated or normal.

In AR patients with elevated eosinophil counts, there were higher temperatures (16.88 ± 9.09) and relative humidity levels (57.75 ± 17.22) compared to those with normal eosinophil counts (temperature: 13.21 ± 10.94 and relative humidity: 52.81 ± 18.24). These differences were statistically significant, with *p* values indicating significance (temperature: *t* = 4.183, *p* < 0.001^∗^; relative humidity: *t* = 2.895, *p*=0.004^∗^). In AR patients with elevated eosinophil counts, there was a notably higher concentration of ozone (O_3_) (116.54 ± 54.92 μg/m^3^) compared to those with normal eosinophil counts (103.73 ± 58.00 μg/m^3^), with a statistically significant difference (*t* = 2.360, *p*=0.018^∗^). However, other pollutants such as PM2.5, PM10, SO_2_, NO_2_, and CO did not exhibit significant differences between the two groups (*p* > 0.05), indicating similar levels regardless of eosinophil counts in AR patients (Table 2). The findings were also visually represented using boxplots to illustrate the distributions of these results (Figure 1).

**Figure 2:**
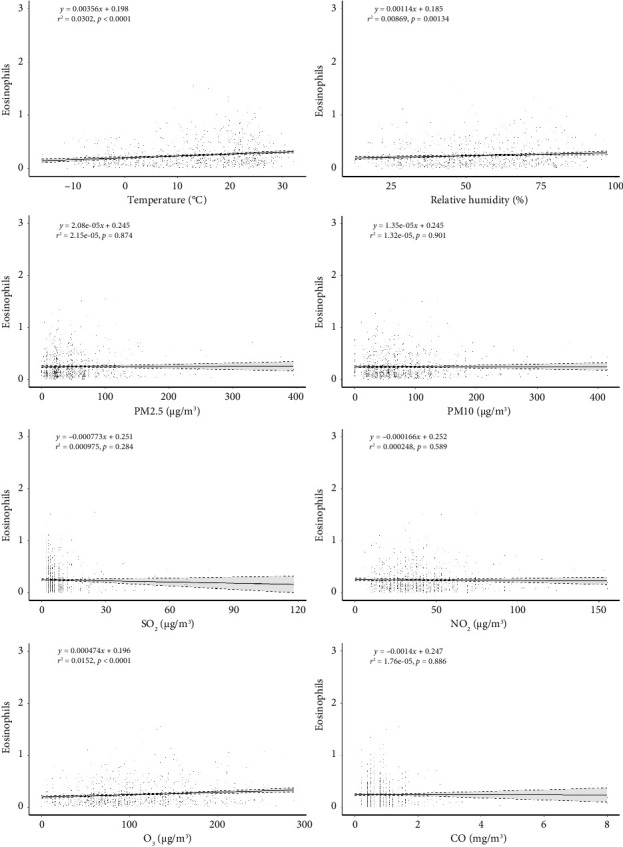
Scatter plot and linear fitting of the relationship between absolute eosinophils count, meteorological factors, and pollutants.

Linear regression analysis revealed a significant positive association between peripheral blood eosinophil count and meteorological factors, including temperature (*β* = 0.003, *p* < 0.05), relative humidity (*β* = 0.001, *p* < 0.05), and O_3_ (*β* = 0.0004, *p* < 0.05) (Figure 2). This suggests that in our sample, an increase in temperature, humidity, and O_3_ levels corresponds to elevated peripheral blood eosinophil counts among patients with AR. The observed coefficients indicate that for every unit increase in temperature, humidity, and O_3_, the peripheral blood eosinophil count tends to increase by 0.003, 0.001, and 0.0004 units, respectively. The coefficient estimates were statistically significant (*p* < 0.05) for all three variables, underscoring the robustness of the observed associations.

## 4. Discussion

Our study investigates the association between meteorological factors, air pollutants, and eosinophil counts in AR patients. As temperature, humidity, and O_3_ levels increase, there is a corresponding rise in peripheral blood eosinophil counts among patients with AR. Our findings corroborate existing research indicating that warmer, more humid climates with higher O_3_ concentrations can lead to increased exposure to allergens and heightened severity of AR symptoms. This is in line with the documented interactions between aeroallergens and air pollutants, wherein pollutants like O_3_ can act as adjuvants, enhancing the immunogenicity of allergenic proteins and exacerbating allergic responses. It is evident that climate change not only affects aeroallergens but also influences air pollutant levels, further complicating the landscape of allergic respiratory diseases. Moreover, the impacts of climate change on airborne allergens are compounded by synergistic effects, emphasizing the complexity of this relationship. In addition, global changes such as desertification, urbanization, and biodiversity loss contribute to variations in airborne allergen levels, which, when combined with climate change, can have varying impacts across different regions and over time [12].

Among the 1080 patients analyzed, nearly 11.85% exhibited elevated eosinophil counts, highlighting the prevalence of this phenomenon within the AR population. We found significant correlations between elevated eosinophil counts and higher temperatures, increased relative humidity, and elevated ozone levels. Among these patients with AR, the days on which elevated eosinophil counts were observed corresponded with significantly higher levels of temperature, relative humidity, and O_3_ concentrations, compared with the days when eosinophil counts remained within the normal range. Linear regression analysis further confirmed these associations, revealing a positive relationship between peripheral blood eosinophil count and temperature, humidity, and O_3_ levels. Our findings suggest that changes in these meteorological factors may influence eosinophil counts in AR patients, indicating a potential environmental influence on allergic responses. However, other pollutants such as PM2.5, PM10, SO_2_, NO_2_, and CO did not exhibit significant differences between the two groups, emphasizing the specificity of the observed associations. Overall, these results underscore the importance of considering environmental factors in the management and treatment of AR, providing valuable insights for future research and clinical practice.

Despite the notable association between eosinophil counts and temperature, humidity, and O_3_ concentration, the lack of significant differences in other pollutants such as PM2.5, PM10, SO_2_, NO_2_, and CO suggests a more nuanced relationship in AR. This implies that while certain environmental factors play discernible roles in affecting eosinophil counts, the impact of other pollutants may be less pronounced or require further investigation in the context of allergic responses. These findings highlight the complexity of environmental influences on eosinophil counts in AR patients, suggesting that specific environmental components may distinctly impact allergic responses and eosinophil counts. Thus, further research into the intricate interplay between environmental factors and immune responses is warranted to gain deeper insights into the mechanisms underlying eosinophil elevation in AR.

Expanding on these observations, insights from environmental and health databases suggest that the nuanced relationship between eosinophil counts and various pollutants might be influenced by factors such as pollutant source, duration of exposure, and individual susceptibility. For instance, while O_3_'s role in exacerbating allergic inflammation is well-documented, the varying degrees of impact from different pollutants may reflect differential mechanisms of action and interactions within the complex immune response pathways [14]. In addition, geographical variations in pollutant concentrations and climate patterns could contribute to the observed differences in eosinophil counts among AR patients. Therefore, a comprehensive understanding of environmental influences on AR necessitates considering not only individual pollutants but also their interactions within specific environmental contexts, paving the way for targeted interventions to mitigate allergic responses and improve respiratory health outcomes.

In recent years, potential mechanisms linking air pollution to AR have been proposed, primarily focusing on exacerbation of inflammation, promotion of oxidative stress, and immune suppression, although precise explanations remain to be definitively confirmed [15]. Multiple studies indicate that air pollution may impair nasal mucosa, induce nasal airway inflammation by mediating responses to nasal allergens, and lead to excessive production of IgE antibodies, rendering the airways hypersensitive to allergen exposure, thus promoting the progression of AR [16, 17]. The interplay between climate change and allergic respiratory diseases, particularly AR, underscores a complex relationship with significant implications for public health. Climate change affects the production and distribution of airborne allergens, exacerbating the burden of AR on vulnerable populations [12]. Studies have extensively documented the influence of climate change on airborne allergens, with rising temperatures, humidity, and CO_2_ concentrations generally associated with increased allergen production [18, 19]. Notably, pollen concentrations have shown an upward trend in response to elevated CO_2_ levels, leading to heightened allergenicity. Similarly, changes in temperature and precipitation patterns affect pollen emission, contributing to variations in pollen seasonality and intensity. However, the impact of climate change on fungal spores remains less explored, with some studies reporting decreasing concentrations attributed to urbanization and changes in land use [20–22]. Nevertheless, projections indicate shifts in the geographic ranges of allergenic species, potentially expanding the scope of AR prevalence. The implications for human health are profound, as climate-induced changes in allergen exposure exacerbate AR symptoms and contribute to the escalation of allergic respiratory diseases. Research suggests a significant increase in sensitization to allergens like ragweed, with projections indicating a doubling of affected individuals in Europe. Moreover, changes in pollen seasonality correlate with a rise in asthma exacerbations and AR-related hospitalizations, particularly in regions experiencing early onset of spring [23].

Climate change not only affects allergen production but also contributes to the activation of allergic responses through mechanisms involving IgE and eosinophils. Elevated CO_2_ levels have been shown to directly increase the allergenicity of pollen, leading to heightened IgE-mediated allergic reactions in susceptible individuals. Moreover, changes in temperature and humidity influence the release of pollen and fungal spores, promoting sensitization and exacerbation of AR symptoms mediated by IgE [24]. Furthermore, climate-induced alterations in atmospheric conditions promote the proliferation of eosinophils, key effector cells in allergic inflammation. Studies have demonstrated a positive association between elevated pollutants levels and peripheral blood eosinophil counts in AR patients, suggesting a potential mechanism through which climate change exacerbates allergic responses. In addition, changes in temperature and O_3_ concentrations correlate with increased eosinophilic inflammation, further amplifying AR severity [25, 26]. Urbanization exacerbates these effects by intensifying exposure to allergens and pollutants, exacerbating allergic inflammation and eosinophil recruitment.

Some limitations of this study should be noted. First, we utilized air monitoring data as a proxy for air pollution exposure for all cases, which may not adequately represent individual-level environmental exposure levels. This potential misclassification of exposure could lead to attenuation of effect estimates. Second, we lacked access to additional individual-level information, including socioeconomic factors and time-activity patterns. For instance, we did not account for time spent indoors and outdoors, as well as the use of air conditioning and heating, indicating that environmental temperature and relative humidity may not reflect true exposure levels. Lastly, due to the absence of allergen information, such as indoor allergens from pets or dust mites, as well as pollen, we were unable to assess allergen exposure for AR. Therefore, future research is necessary to confirm the impact of air pollution and meteorological factors on eosinophil counts in AR patients by collecting more comprehensive data [27, 28].

## 5. Conclusion

In summary, our study highlights the link between meteorological factors and peripheral blood eosinophil count in AR patients. Among 1080 patients, nearly 12% had elevated eosinophil counts, correlating with higher temperatures, relative humidity, and O_3_ levels. Linear regression analysis confirmed these associations. Our findings suggest that environmental factors may influence eosinophil counts in AR, emphasizing the need to consider such factors in treatment strategies.

## Figures and Tables

**Table 1:** Baseline of clinical characteristics for patients with AR.

Characteristics	Number
Age (mean ± SD)	32.80 ± 19.70
Male (*n*, %)	605 (56.02%)
Female (*n*, %)	475 (43.98%)
Peripheral blood eosinophils count (mean ± SD)	0.25 ± 0.23
Elevated eosinophils count (*n*, %)	128 (11.85%)
Normal eosinophils count (*n*, %)	952 (88.15%)

**Table 2:** The association between elevated eosinophils in AR patients with meteorological factors and pollutants (mean ± SD).

Characteristics	Elevated eosinophils counts	Normal eosinophils counts	t	*p*
Temperature (°C)	16.88 ± 9.09	13.21 ± 10.94	4.183	< 0.001⁣^∗^
Relative humidity (%)	57.75 ± 17.22	52.81 ± 18.24	2.895	0.004⁣^∗^
PM2.5 (μg/m^3^)	50.43 ± 49.50	49.48 ± 49.31	0.205	0.837
PM10 (μg/m^3^)	76.41 ± 55.13	77.81 ± 59.95	0.252	0.801
SO_2_ (μg/m^3^)	6.12 ± 6.49	6.83 ± 9.02	0.859	0.391
NO_2_ (μg/m^3^)	38.78 ± 19.73	38.49 ± 21.19	0.145	0.885
O_3_ (μg/m^3^)	116.54 ± 54.92	103.73 ± 58.00	2.360	0.018⁣^∗^
CO (mg/m^3^)	0.81 ± 0.48	0.80 ± 0.66	0.219	0.826

⁣^∗^*p* < 0.05.

## Data Availability

The data underlying the findings of this study are available from the corresponding author upon reasonable request.
